# Patient-Reported Barriers to Mammography Screening Among Women at a Community Health Clinic in Southeastern Michigan: A Cross-Sectional Pilot Study

**DOI:** 10.7759/cureus.115716

**Published:** 2026-09-03

**Authors:** Miranda Goodson, Karen Abdelmalek, Dina Bayachou, Rhea Sehgal, Dea Kanani, Whitney Bowyer, Alexis Kralovich, Anna Lundh, Sarah Qureshi, Nina Agemy, Laura J Benjamins

**Affiliations:** 1 Medicine, Wayne State University School of Medicine, Detroit, USA; 2 Pediatrics, Wayne State University School of Medicine, Detroit, USA

**Keywords:** breast cancer, gender health, i am interested in women's health, mammography, michigan, preventive mammogram, public health education, screening mammogram, women, women and health

## Abstract

Background: The objective of this cross-sectional pilot study was to explore patient-reported barriers to mammography screening and women's knowledge, attitudes, and behaviors regarding mammography among women receiving care at a university-affiliated community health clinic in Southeastern Michigan (SEM).

Methods: A 47-item investigator-developed exploratory questionnaire informed by the Health Belief Model (HBM) constructs was distributed via Qualtrics from August to December 2025 to women receiving care at a local community health clinic. The questionnaire assessed the participants' awareness, perceptions, attitudes, and comfort regarding mammogram screening. Descriptive statistics were calculated for completed questionnaires, and patient-reported barriers were categorized using prespecified response categories.

Results: Twenty-one adult women aged 18 and older completed the questionnaire. Findings demonstrated that most participants were unaware of affordable or free mammogram services (81%, n = 17), while 28.6% (n = 6) reported language as a barrier in healthcare. The perception of mammography costs also varied across insurance groups.

Conclusion: This community-focused pilot study provides preliminary insights into the perceptions, beliefs, attitudes, experiences, and potential barriers related to mammography screenings among women receiving care at a community health clinic in SEM. Although these preliminary findings should be interpreted with caution due to their exploratory study design and small convenience sample, they may inform future community-oriented research examining barriers to mammography screening and the development of region-specific educational and outreach strategies.

## Introduction

Breast cancer remains the most commonly diagnosed cancer among American women [1]. Mammography is the most widely used screening modality for breast cancer and can detect early-stage, non-palpable malignancies, often before symptoms develop [1]. Current US Preventive Services Task Force guidelines recommend biennial screening mammography for average-risk women aged 40-74 years [2]. Widespread adoption of mammography has contributed to a substantial decline in breast cancer mortality in the United States between 1987 and 2017 [3].

Despite these well-established benefits, screening adherence remains suboptimal. National public health data indicate that only about 65% of US women are up to date with recommended mammography screening guidelines [4]. Numerous factors contribute to the underutilization of mammography, including lack of health insurance or underinsurance, out-of-pocket costs, limited access to healthcare facilities, transportation difficulties, competing work or caregiving responsibilities, and limited knowledge about screening [5,6]. Psychological and social barriers, such as fear of pain or diagnosis, mistrust of the healthcare system, and lack of an adequate healthcare provider recommendation, further reduce screening participation [5,7].

Importantly, mammography compliance varies significantly by race and socioeconomic status. Women of color are less likely to receive routine mammograms than White women, despite having similar or higher rates of breast cancer incidence and higher mortality rates [8]. Structural inequities, including neighborhood-level resource disparities, reduced access to preventive care, implicit bias in healthcare delivery, and historical and ongoing mistrust of medical institutions, contribute to these differences [5,9].

The health belief model (HBM) provided the conceptual framework for examining preventive health behaviors in this study. This model contextualizes health behaviors through an analysis of how individuals perceive a health threat and make decisions to prevent or respond to that threat [10]. The HBM constructs considered in this study included perceived barriers, perceived benefits, cues to action, and self-efficacy [10]. The objective of this cross-sectional pilot study was to explore patient-reported barriers to mammography screening and women's knowledge, attitudes, and behaviors regarding mammography among women at a university-affiliated community health clinic in Southeastern Michigan (SEM).

## Materials and methods

This exploratory cross-sectional pilot study was conducted at a university-affiliated community health clinic in SEM from August to December 2025 (Figure 1). Women aged 18 years or older were invited to voluntarily complete the anonymous survey either through direct recruitment by a research team member during a clinic visit or by accessing the survey link or QR code provided on study flyers distributed throughout the clinic. Eligibility criteria included being a US citizen, identifying as a woman, being 18 years of age or older, and receiving care at the participating clinic. Women younger than the recommended age for routine screening were included because the broader pilot sought to explore mammography knowledge, attitudes, and barriers across the clinic population, including perspectives relevant to future screening. The number of eligible patients who were approached but declined participation was not recorded; therefore, a recruitment response rate could not be calculated. Surveys were available in English, Arabic, and Spanish. The English questionnaire was translated to Arabic and Spanish by bilingual research team members with relevant medical and cultural backgrounds to support cultural and linguistic appropriateness. Prior to participation, respondents reviewed an IRB-approved study information sheet, and completion of the survey indicated consent to participate. The study protocol (IRB-24-07-6999) was reviewed and approved by the Wayne State University Institutional Review Board. 

**Figure 1:**
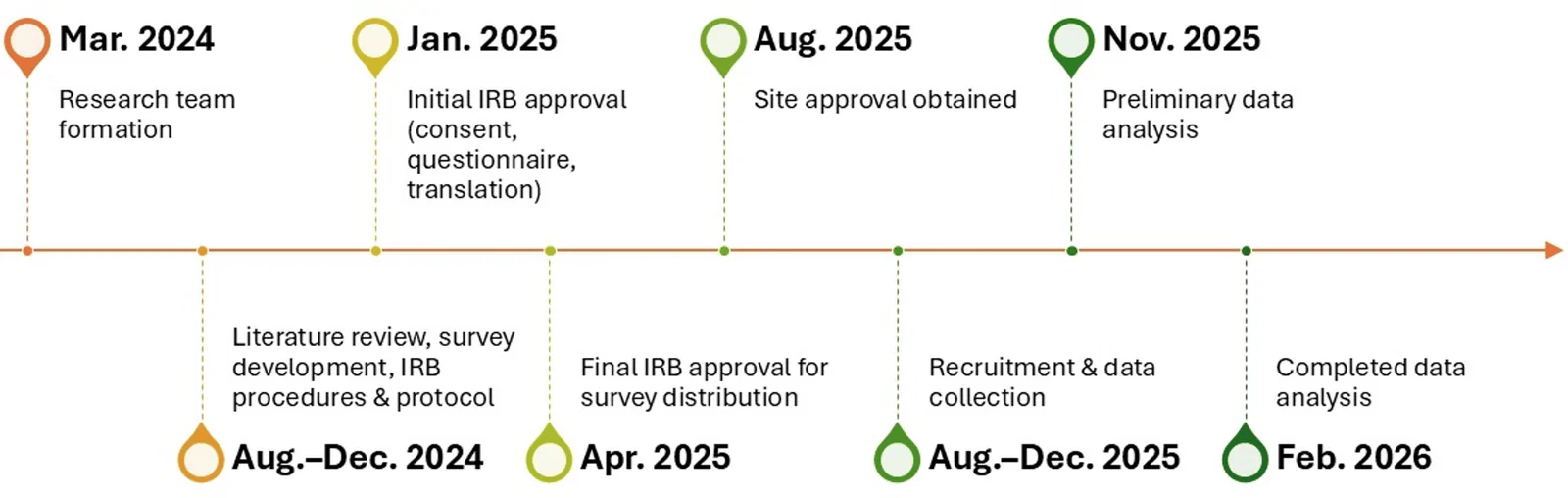
Study timeline Colors represent the sequential study phases: orange = study development; yellow = IRB approval process; light green = site access and data collection; dark green = data analysis Image created using Microsoft PowerPoint (Microsoft® Corp., Redmond, WA)

The 47-item descriptive questionnaire collected demographic and background information and assessed mammography history, screening behaviors, awareness, perceived cost, healthcare access, and patient-reported barriers, as seen in Appendix 1. Although there are validated mammography screening instruments available, none completely aligned with the exploratory objectives and specific community context of this pilot study. Questionnaire items were developed by the research team based on the study objectives, relevant mammography screening literature, HBM constructs, and issues considered pertinent to the clinic population. The questionnaire was developed and distributed using Qualtrics (Qualtrics, Provo, UT) through the 2024-2025 platform version provided by Wayne State University School of Medicine. Data were collected, organized, and analyzed in Microsoft Excel (Microsoft® Corp., Redmond, WA). 

Survey questions were optional, allowing the participants to omit questions based on their comfort level. Participants who achieved 100% survey progress in Qualtrics were included in the analytic sample. Participants who did not progress through the entire survey or had insufficient responses to barrier-related questions were excluded. A total of 35 survey attempts were recorded, with 21 participants meeting the inclusion criteria for this study's analysis, and 14 excluded due to incomplete survey attempts (Figure 2). Missing responses among the included 21 participants were retained and reported as "no response", where applicable, instead of being excluded from the overall analytic sample. 

**Figure 2:**
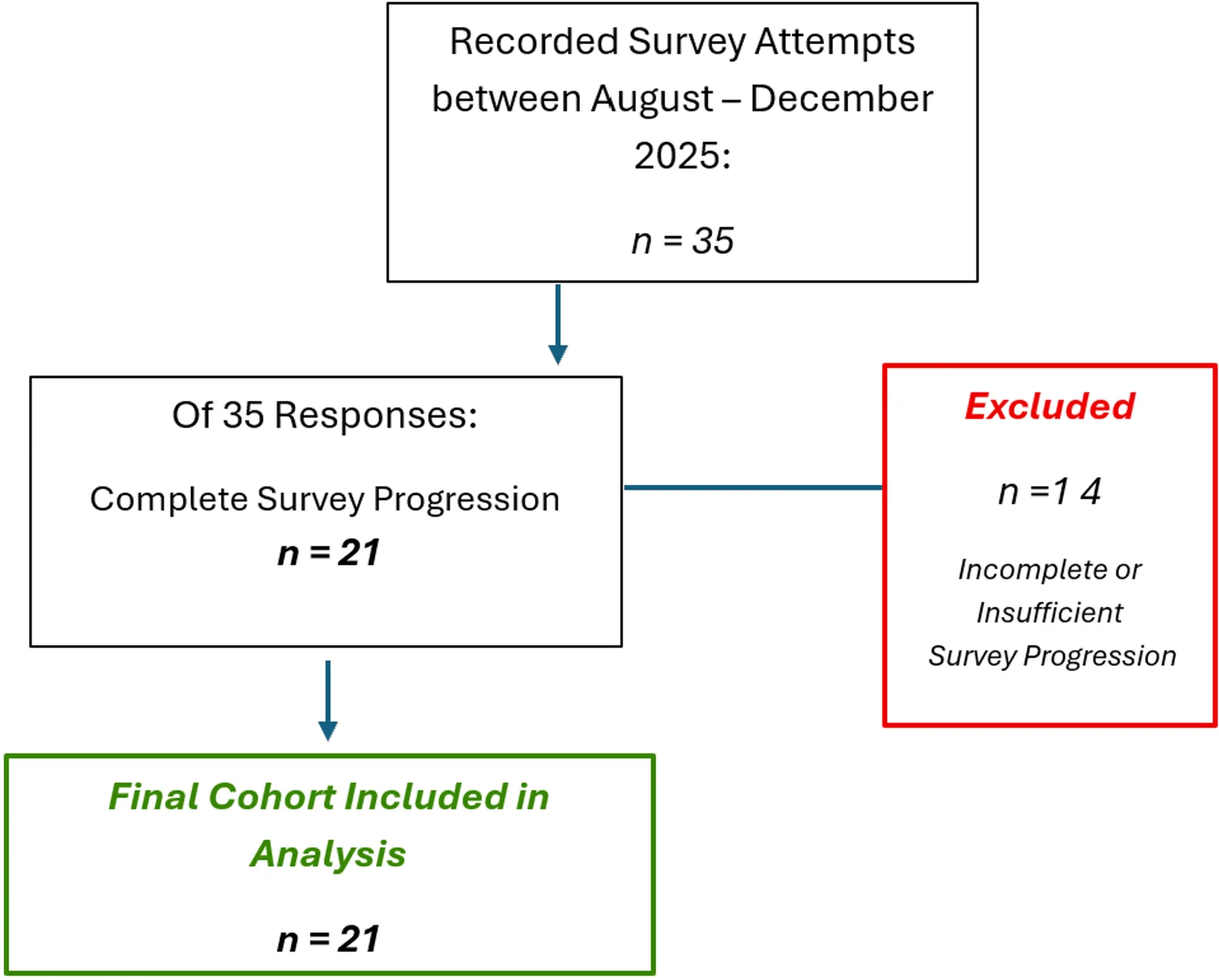
Participant inclusion flowchart Image created using Microsoft PowerPoint (Microsoft® Corp., Redmond, WA)

Descriptive statistics were used to summarize participant responses. For any subgroup analyses, percentages were calculated using the corresponding subgroup denominator. A summary of patient-reported barriers was obtained using prespecified response categories included throughout the questionnaire. Barrier-related items were summarized using predefined response options, so no qualitative coding was performed. Since this was an exploratory pilot study designed to collect preliminary descriptive data, rather than test a hypothesis, no formal power calculation was conducted.

## Results

All participants (Table 1) aged 36-65 years self-reported receiving at least one mammogram in their lifetime, compared to only one participant in the 25-35 years of age group, who reported a prior mammogram (Table 2).

**Table 1:** Participant demographics (n = 21) Participant demographic characteristics, including race/ethnicity, age, and employment status. Values are expressed as the number of participants (n) and the percentage of the analytic sample (%).

Characteristic	n	%
Race/Ethnicity		
Middle Eastern/North African (MENA)	13	61.9%
Asian	3	14.3%
White or Other	4	19.0%
Black/African American	1	4.8%
Age		
18-24 years	1	4.8%
25-35 years	8	38.1%
36-55 years	9	42.9%
56-65 years	3	14.3%
Employment status		
Unemployed	13	61.9%
Full time	6	28.6%
Part time	2	9.5%

**Table 2:** Mammography behaviors by age group (n = 21) Age-stratified mammography history and self-reported likelihood of routine screening. Values are expressed as the number of participant responses and the percentage within each age group, n (%).

Age group	Ever had mammogram	Never had mammogram	Extremely likely routine screening
18-24 years	0 (0.0%)	1 (100.0%)	0 (0.0%)
25-35 years	1 (12.5%)	7 (87.5%)	3 (37.5%)
36-55 years	9 (100.0%)	0 (0.0%)	7 (77.8%)
56-65 years	3 (100.0%)	0 (0.0%)	2 (66.7%)

More than half of respondents, 57.1% (n = 12), reported being extremely likely to obtain annual mammograms, while 42.9% (n = 9) reported being extremely likely to utilize affordable or free mammography services if available. Although many participants indicated a willingness to use accessible screening services, 81.0% (n = 17) reported being unaware of affordable or free mammography services. Language was reported as a healthcare barrier by 28.6% (n = 6) of participants (Table 3).

**Table 3:** Mammography behaviors and barriers (n = 21) Participants reported mammography screening behaviors, awareness of affordable or free services, and language barriers to navigating healthcare. Values are expressed as the number of participants (n) and the percentage of the analytic sample (%).

Survey findings	n	%
Likelihood of annual mammograms		
Extremely likely	12	57.1%
Somewhat likely	4	19.0%
Neither likely nor unlikely	4	19.0%
No response	1	4.8%
Likelihood of using affordable/free services		
Extremely likely	9	42.9%
Somewhat likely	4	19.0%
Neither likely nor unlikely	3	14.3%
Somewhat unlikely	1	4.8%
Extremely unlikely	3	14.3%
No response	1	4.8%
Awareness of affordable/free services		
Yes	3	14.3%
No	17	81.0%
No response	1	4.8%
Language barrier		
Yes	6	28.6%
No	15	71.4%

Other barriers assessed included psychological and logistical factors, with each barrier reported by fewer than 20% of participants (Table 4).

**Table 4:** Additional patient-reported barriers to mammography and healthcare (n = 21) Additional psychological and logistical barriers were self-reported by participants, including prior healthcare experiences, embarrassment or exposure during mammography, fear of medical procedures, childcare responsibilities, general fear or anxiety about medical care, and previous healthcare-related trauma. Values are expressed as the number of participants (n) and the percentage of the analytic sample (%).

Additional barriers	n	%
Negative prior healthcare experiences	4	19.0%
Embarrassment/exposure during mammography	4	19.0%
Fear of medical procedures	3	14.3%
Childcare responsibilities	2	9.5%
General fear/anxiety about medical care	2	9.5%
Previous healthcare-related trauma	1	4.8%

Descriptive differences in perception of mammography costs were observed across insurance categories represented in this sample. All Medicare participants identified the perceived cost as $0, compared to only 40.0% (n = 2) of Medicaid participants and 22.2% (n = 2) of privately insured participants. Most privately insured participants (66.7%, n = 6) believed mammography would cost more than $100 (Table 5).

**Table 5:** Perceived mammogram cost by insurance type (n = 21) Perception of mammography cost stratified by insurance type based on participant self-report. Values are expressed as the number of participant responses and percentage within each insurance group, n (%).

Insurance Type	n	$0	<$100	>$100	No response
Private	9	2 (22.2%)	1 (11.1%)	6 (66.7%)	0
Medicare	5	5 (100.0%)	0	0	0
Medicaid	5	2 (40.0%)	1 (20.0%)	2 (40.0%)	0
Uninsured	2	0	1 (50.0%)	0	1 (50.0%)

## Discussion

Our pilot exploratory study identified several patient-reported elements pertinent to mammography screening amongst women receiving care at a community health clinic in SEM. Self-reported data demonstrated that 81.0% of participants were unaware of affordable or free mammography services, 28.6% reported language as a healthcare barrier, and perception of mammography cost differed across insurance types (Tables 3, 5). Despite these reported barriers, participants expressed general willingness to engage in mammography screening, with 57.1% of participants reporting that they were extremely likely to get their annual mammograms and 42.9% reporting that they were extremely likely to utilize affordable or free mammography services if available. With most participants identified as Middle Eastern/North African (MENA) women, our findings should be interpreted in this context. Previous research focused on Arab American women in metropolitan Detroit, the largest city in SEM, reported that 61.9% adhered to biennial mammography screening recommendations [11]. Similarly, 57.1% of participants in our cohort reported being extremely likely to obtain routine mammography screening (Table 3); of the 12 participants with this response, eight identified as MENA. Although direct comparisons cannot be made due to the small convenience sample and differences in outcome measures, these findings provide regional context for screening attitudes reported in our cohort.

The findings that 81.0% of participants in our study were unaware of affordable or free mammography services may represent an opportunity for more community-partnered education and outreach initiatives (Table 3). The Community Preventive Services Task Force recommends the addition of community health worker interventions (CHWI) to increase breast cancer screening. Across multiple studies, CHWI increased screening participation by 12.7%, with greater improvements when interventions addressed educational and access barriers, including the addition of translation services, resulting in a 30.2% increase in participation [12]. In the present study, 28.6% of participants reported language as a barrier to receiving healthcare, indicating that language-related obstacles were present within this cohort (Table 3). Previous CHWI in SEM have demonstrated improvements in breast cancer screening knowledge among Arab American women (p < 0.001) [13]. These interventions provided educational materials in participants' preferred language, with 68% of the Arab American participants requiring translated materials to participate [13].

Screening mammograms are generally covered at no out-of-pocket cost for Medicare beneficiaries and for most individuals with private insurance and Medicaid under the Affordable Care Act (ACA) [2,14]. Among the five Medicare participants, all reported a perceived mammography screening cost of $0 (Table 5). In contrast, only 40.0% of Medicaid participants and 22.2% of privately insured participants believed screening mammograms cost $0, while 66.7% of privately insured participants believed they cost more than $100 (Table 5). These findings may reflect differences in participant understanding of mammography costs and insurance coverage rather than actual out-of-pocket screening costs. Given the small subgroup sizes, these findings are presented descriptively and should not be interpreted as representative of differences between insurance groups. Previous studies have shown that many patients remain unaware of the ACA's zero cost-sharing requirement and often confuse screening with diagnostic mammography, which may involve additional out-of-pocket costs [2,14]. Indirect costs, including transportation, childcare, and time away from work, may further reduce screening participation [15]. Increasing awareness of insurance coverage and community-based screening programs may help reduce these misconceptions and improve mammography utilization [12,16].

The constructs of the HBM provided a theoretical framework for interpreting these descriptive findings. Participant-reported language challenges and cost misconceptions may reflect perceived barriers. Participants' willingness to obtain mammography and utilize affordable or free screening services may be related to perceived benefits and self-efficacy. Awareness of affordable screening programs, translation services and materials, and CHWI may function as cues to action [10].

This study has several limitations. The small sample size, recruitment from a single community healthcare clinic, and use of convenience sampling limit the generalizability of the findings and present the possibility of selection bias. The developed questionnaire and translated version were not previously validated. This may limit measurement reliability and comparability with studies using validated instruments. Inclusion of participants younger than the recommended age for routine breast cancer screening may have influenced screening-specific responses, while the reliance on self-reported data has the potential for recall and social desirability bias. The number of eligible patients who declined participation was not recorded; therefore, the recruitment response rate could not be calculated. Furthermore, no participants were older than 65 years of age. Subgroup analyses contained even smaller sample sizes, especially between insurance and demographic categories, and should be considered as descriptive only, since the study was not designed or powered to evaluate associations between participant characteristics and mammography behaviors.

As the first phase of an ongoing research initiative, these findings will be used to refine future iterations of the questionnaire by optimizing survey length, improving completion rates, expanding questions about mammography behaviors and cost perceptions, and incorporating additional cultural and socioeconomic items. The next version of our questionnaire will go through a formal evaluation and implement standardized procedures for monitoring recruitment and nonresponse.

## Conclusions

This community-based pilot study serves as a foundational step toward understanding mammography screening experiences among women receiving care at a community health clinic in SEM. The findings include self-reported language barriers, limited awareness of affordable and accessible mammography screening services, and possible misconceptions regarding mammography costs and insurance coverage within this study population. Given the exploratory design, small convenience sample, and single-site recruitment, these findings should be interpreted with caution and are not intended to be generalized beyond this study's population. Future studies with larger and more representative samples across SEM can further evaluate these barriers and inform culturally responsive educational initiatives and community-centered interventions aimed at improving mammography utilization.

## Appendices

Appendix 1: Mammography study questionnaire

Background

What is your age? (select one)

18-24

25-35

36-55

56-65

66-75

76+

What is your race? (select one)

American Indian or Alaska Native

Asian

Black or African American

Middle East or North Africa

Native Hawaiian or Other Pacific Islander

White

Prefer not to say

Other (please specify below): __________

What is your gender identity? (select one)

Male

Female

Transgender (a person whose gender identity does not correspond with the sex registered for them at birth)

Non-binary/third gender (genders that are not exclusively male or female)

Prefer not to say

Other (please specify below): __________

What is your sexual orientation? (select one)

Straight

Gay/Lesbian

Bisexual

Prefer not to say

Other (please specify below): __________

What is your highest level of education? (select one)

Less than a high school diploma

High school diploma

GED

Associates

Trade School

Bachelor's

Masters

Doctorate

What zip code do you live in? (specify below)

Medical History

Q1. Have you ever received a mammogram? (select one)

Yes

No

If "Yes":

When was your last mammogram? (select one)

Within the last year

1-2 years ago

3-5 years ago

5-10 years ago

10+ years ago

I don't remember

Do you have a primary care physician or OB/GYN that you see regularly? (select one)

Yes

No

If "Yes":

When was your last appointment? (select one)

Less than a year ago

1-2 years ago

3-5 years ago

5+ years ago

I don't remember

Do you have a family history of breast cancer? (select one)

Yes

No

Do you have a family history of ovarian cancer? (select one)

Yes

No

Has a family history of breast cancer or ovarian cancer impacted your decision to get a mammogram? (select one)

I am more likely to get one

I am less likely to get one

I do not feel impacted by it

Have you ever been diagnosed with breast or ovarian cancer? (select one)

I have been diagnosed with breast cancer

I have been diagnosed with ovarian cancer

I have been diagnosed with both breast and ovarian cancer

I have never been diagnosed with breast or ovarian cancer

Employment/Coverage

Are you currently employed?

Full Time

Part Time

No

Are you currently receiving government assistance? (SNAP, EBT, WIC, etc)

Yes

No

If "Yes":

Select which form(s) of government assistance you are currently receiving (select all that apply)

SNAP

EBT

WIC

Other, Explain: __________

Describe your health coverage (select one)

Uninsured

Private

Medicare

Medicaid

Veterans Affairs (VA)

Prefer Not To Say

Barriers to Healthcare

Transportation

How long does it take you to get to your doctor's office?

less than 10 minutes

between 10 - 30 minutes

between 30 - 60 minutes

more than an hour

Not applicable

How do you usually get to the doctor?

Car

Public Transport (Bus/Train/Trolly)

Ride Share (Uber/Lyft)

Ride from Family/Friend

Bike

Walk

Other: __________

Not applicable

Have you ever worried about having a reliable way to get to and from your medical appointment?

Never

Sometimes

About half the time

Most of the time

Always

Has transportation hindered you from getting a mammogram?

Yes

Maybe

No

Money

Have any of these stopped you from getting a mammogram?

Out-of-pocket cost

Co-pay or deductible

Lost wages from taking time off work

Other: __________

None of the above

How much do you think a mammogram might cost with health insurance? (select one)

$0

Less than $100

More than $100

Other

Are you worried about having trouble understanding or speaking the language at your appointment?

Yes

No

Does fear of going to the doctor stop you from getting care?

Yes

No

Is finding childcare a problem when you go to medical appointments?

Yes

No

Does anxiety about medical issues keep you from seeking care?

Yes

No

Does fear of medical procedures stop you from getting them?

Yes

No

Have negative past experiences with healthcare made you avoid future care?

Yes

No

Have you experienced trauma that now makes you nervous or unsure about going to the doctor?

Yes

No

Does the idea of being undressed or exposed stop you from getting a mammogram?

Yes

No

For each of the following items, rate how important they are to you when it comes to getting a mammogram.

Response options: Not at all important | Slightly important | Moderately important | Very important | Extremely important

Access to transportation

Your distance to the clinic

Cost of procedure

Medical Insurance

Concerns about pain during the procedure

Language barriers

Breast cancer stigma within the community

Do you regularly check your breasts for any lumps or changes?

Yes

No

If "Yes":

If so, how often?

About once a week

About once a month

About once every couple of months

About once a year

Attitudes

How likely are you to get a yearly mammogram to check for breast cancer?

Extremely unlikely

Somewhat unlikely

Neither likely nor unlikely

Somewhat likely

Extremely likely

How likely are you to get a mammogram if you notice something concerning, like a lump or other symptoms?

Extremely unlikely

Somewhat unlikely

Neither likely nor unlikely

Somewhat likely

Extremely likely

How would you rate your understanding of what mammograms are and what they check for?

Extremely bad

Somewhat bad

Neither good nor bad

Somewhat good

Extremely good

Do you know someone who has had a mammogram?

Yes

No

How comfortable are you talking about getting a mammogram with your doctor?

Extremely uncomfortable

Somewhat uncomfortable

Neither comfortable nor uncomfortable

Somewhat comfortable

Extremely comfortable

Are there any other things not mentioned in this survey that might affect whether you're willing or able to get a mammogram?

Affordable Services

Do you know about any affordable or free mammogram services available in your area?

Yes

No

If you were given information about a free or affordable mammogram service, how likely would you be to use it?

Extremely unlikely

Somewhat unlikely

Neither likely nor unlikely

Somewhat likely

Extremely likely

Resources

Here's some information about free mammograms available in Detroit: HOPE is partnering with Wayne State Mobile Health Units and Ascension Michigan to provide more access to healthcare resources. Ascension Michigan will offer mammograms to screen for breast cancer. These mobile services will be available once a month with no eligibility requirements. To get a mammogram with Ascension Michigan, you need to register by calling 313-494-4600. The service is available from 9 a.m. to 3:45 p.m. on Mondays at various locations.

Westside Food Center (April 15): 1300 Oakman Boulevard, Detroit

Eastside Food Center (May 20): 9151 Chalmers Street, Detroit

Westside Food Center (June 17): 1300 Oakman Boulevard, Detroit

Inkster Food Center (July 15): 759 Inkster Road, Inkster

Eastside Food Center (August 19): 9151 Chalmers Street, Detroit

Westside Food Center (Sept. 16): 1300 Oakman Boulevard, Detroit
